# Heart diseases, hypertension and effects of antihypertensive medications: Is hypertension a true risk factor of heart diseases?

**DOI:** 10.3389/fpubh.2022.929840

**Published:** 2022-10-31

**Authors:** Kazumitsu Nawata

**Affiliations:** Hitotsubashi Institute for Advanced Study (HISA), Hitotsubashi University, Tokyo, Japan

**Keywords:** heart disease, cardiovascular disease, hypertension, antihypertensive medication, cholesterol

## Abstract

**Background:**

Heart diseases (HD) are the leading cause of deaths in the world. Many studies have been done on the relationships among hypertension, HD and antihypertensive medications. Most of the studies find that hypertension is a significant risk factor of HD, but there are some studies in which hypertension is not a risk factor. As antihypertensive medications are routinely prescribed to prevent HD, it is necessary to evaluate the effects of these and other risk factors of HD.

**Data and methods:**

The relationship between hypertension and HD was analyzed using 6,773,464 medical checkups obtained from the JMDC Claims Database obtained from January 2005 to September 2019. Factors potentially affecting HD, including blood pressures (BP) and usage of antihypertensive medications, were evaluated using 2,861,769 observations. To avoid the causality problem, probit models were used to analyze the probability of an individual who had no history of HD at year t developing HD by year *t* + 1.

**Results:**

A positive relation between systolic blood pressure (SBP) and HD was found in the equation without any other covariates. However, the significant relation between HD and BP disappeared when the models contained various other factors as covariates. When a 10-year age or longer interval was used in the model, a positive relation between the two variables was found, suggesting that SBP works as a proxy variable. Taking antihypertensive medications greatly increases the probability of developing HD in the next year. Higher levels of cholesterols decrease the probability of developing HD.

**Conclusion:**

Unlike many previous studies, no significant relationship between HD and hypertension was found in the models containing multiple covariates. The accepted relation might actually be spurious, and it is important to select covariates carefully. Taking antihypertensive medications appears to increase the probability of developing HD in the next year, suggesting the need for further research and greater caution in the use of antihypertensive medications.

## Introduction

Ischaemic heart disease (IHD) is the leading cause of deaths worldwide. The World Health Organization (WHO) (1) estimated that in 2019, 8.9 million or 16% of total deaths worldwide were caused by IHD. In the United Sates, the Centers for Disease Prevention and Control (CDC) (2) reported that heart diseases (HD) were the leading cause of death, responsible for 655,381 deaths or 23.1% of all 2,839,205 deaths in 2018. The American Heart Association (AHA) (3) reported that coronary heart disease (CHD) caused 365,744 or 13% of total deaths in 2018. The estimated annual incidents of heart attack were 605,000 new attacks and 200,000 recurrent attacks from 2005 to 2014, and the estimated annual average cost of HD in 2016 and 2017 was $219.6 billion. However, awareness of the importance of heart health may be declining: Cushman (4) reported that in the U.S., awareness that HD is the leading cause of death among women declined from 65% in 2009 to 44% in 2019.

In Japan, HD was the second-leading cause of death and caused 205,596 deaths or 15.0% of total deaths of 1,372,755 in 2020 (5). The estimated number of HD patients was 1,782 thousand (64.0 thousand inpatients) on a survey day in 2017 (6). The medical costs of HD were 2.09 trillion yen or 4.5% of total medical expenditures in 2019 (7). Comorbidities make this situation more urgent. Liang et al. (8) concluded in a meta-analysis that CHD is a risk factor for poor prognosis in patients with COVID-19. Moreover, Lehmann et al. (9) pointed out that large numbers of cardiac patients suffer from depression and emphasized the importance of screening for suicidal ideation in clinical practice.

The prevention of HD is a very important issue. High blood pressure (BP) or hypertension is considered a major risk factor of HD (10, 11), and many studies have been done on this issue. BP is measured as systolic blood pressure (SBP, mmHg) and diastolic blood pressure (DBP, mmHg). Under the “Seventh report of the joint national committee on prevention, detection, evaluation, and treatment of high blood pressure” (JNC7) guideline settled in 2003, the criteria for hypertension were 140/90 mmHg: i.e., SBP ≥140 or DBP ≥90 mmHg (12, 13). In 2017 the American College of Cardiology (ACC), AHA and other organizations (14) presented a new guideline (2017ACC/AHA guideline) for hypertension, lowering the criteria for hypertension to 130/80 mmHg (SBP ≥130 or DBP ≥80 mmHg). However, other organizations such as the American Diabetes Association (15), European Society of Cardiology and European Society of Hypertension (ESC/ESH) (16), Hypertension Canada (17), and the Japanese Society of Hypertension (18) have maintained the conventional 140/90 mmHg criteria. Sepanlou et al. (19) reported that in Iran, the sex- and age-standardized prevalence of hypertension was 22.3% and 36.5% under the JNC7 guideline and the 2017 ACC/AHA guideline, respectively. For the potential implications of the two definitions, see Goupil et al. (20).

The basis of the 2017 ACA/AHA guideline is that hypertension is a major risk factor for HD; that is, the risk of having HD increases as BP increases. Nonetheless, various studies on the relationship between hypertension and HD have raised questions regarding this medical axiom as described the next section. In the present study, the relationships among BP, HD and antihypertensive medications are analyzed using the JMDC Claims Database, which include 13,157,681 medical checkups performed on 3,233,271 individuals in Japan. Other risk factors of HD are evaluated as covariates in several different models.

## Previous studies

Many studies concerning the relationships between BP and HD have been performed. “Cardiovascular Disease” (CVD) was frequently used in these studies. According to the National Heart, Lung and Blood Institute (21), CVD is the term for all types of diseases that affect the heart or blood vessels, including CHD, which can cause heart attacks, stroke, congenital heart defects and peripheral artery disease. “CVD” is used based on the original studies.

The Framingham Heart Study (22) has been continuously conducted since 1948 in Framingham, Massachusetts. National Institute of Health (23) states that most CVD is caused by modifiable risk factors like smoking, high BP, obesity, high cholesterol levels, and physical inactivity.

Lewington et al. (24) conducted a meta-analysis obtained from the results of 61 prospective studies. This study analyzed the data of 12.7 million person-years and reported 56,000 vascular mortalities including 34,000 mortalities caused by IHD. They also reported that IHD mortalities increased as SBP and DBP increased in all age cohorts.

The Systolic Blood Pressure Intervention Trial (SPRINT) (25) was a trial enrolling 9,361 participants with SBP ≥130 mmHg and known cardiovascular risks but not diabetes. This study's findings were given substantial weight in the 2017 ACC/AHA guideline. The participants were randomly divided into two groups. One was an intensive treatment group with an SBP target <120 mmHg, and the other was the standard treatment group aiming for the JNC7 guideline of SBP <140 mmHg. The former and latter groups contained 4,678 and 4,683 participants, respectively. The enrollment periods were from 2010 to 2013. They found that the rates of fatal and nonfatal major CVD and of death from any cause in the intensive treatment group were lower than those in the standard treatment group.

Ettehad et al. (26) performed a meta-analysis using 123 studies selected from 11,428 studies that focused on the effects of BP from 1966 to 2015. The data of 613,815 individuals were used in the analysis. They concluded that treatments for lowering BP reduced the CVD risk and that the mortality rates from all causes were reduced by 13% when SBP was lowered by 10 mmHg.

Rapsomaniki et al. (27) analyzed 2.25 million people from 1977 to 2010 using CALIBER (CArdiovascular research using LInked Bespoke studies and Electronic health Records). In the analysis, 83,098 cases of first-time CVD were observed. The authors estimated that the lifetime risk of CVD for hypertensive individuals at age 30 was 63.3%, much higher than that (46.1%) of normotensive individuals.

Yusuf et al. (28) conducted the Prospective Urban Rural Epidemiology (PURE) study. The PURE study is a multinational and prospective cohort study, and its dataset was obtained from 21 high-income, middle-income, and low-income countries. Associations for 14 potentially modifiable risk factors with mortality and CVD were examined in 155,722 participants without a prior history of CVD. The enrollment period was between January, 2005 and December, 2016. The Cox frailty models were used in the study. They reported that hypertension and education had extensive global effects.

In Japanese subjects, Fujiyoshi et al. (29) evaluated the relation between BP and CVD using a dataset of 63,309 individuals in Japan using age- and gender-based cohort analysis. Among their participants, 1,944 CVD mortalities occurred within 10.2 years, and analysis revealed a positive relation between CVD and BP. Asayama et al. (30) examined the mortality risk due to CVD using a dataset of 39,705 participants from 6 selected cohorts. The Cox proportional hazard model was used in the analysis. They reported that the CVD mortality risk became higher for individuals without treatment even if the effects of various factors of participants and cohorts were removed. On the other hand, no clear relation between hypertension stages and CVD mortality risk was observed for males under treatment for hypertension. Honda et al. (31) analyzed 2,462 residents aged 40–84 for 24 years using the Cox proportional hazard model obtained from the Hisayama study. They reported that age, gender, SBP, hemoglobin A1c (HbA1c), low-density lipoprotein cholesterol (LDL), high-density lipoprotein cholesterol (HDL), smoking, and daily exercise were predictive factors of CVD.

Most of these studies indicated that hypertension (especially high SBP) is a risk factor of CVD. Muntner et al. (32) noted that the 2017 ACC/AHA guideline would increase medication usage and reduce the prevalence of CVD. Fuchs and Whelton (33) demonstrated that, among the risk factors for CVD, hypertension had the strongest evidence for causation.

On the other hand, the Action to Cardiovascular Risk in Diabetes (ACCORD) study (34), which involved 4,733 persons with type 2 diabetes, reported that lowering the SBP below 120 mmHg did not reduce major CVD events or death rates compared to the cases in which the SBP was lowered below 140 mmHg. Note that the SPRINT study used the same framework as this ACCORD study.

Saiz et al. (35) performed a systematic review of databases for six randomized controlled trials (RCT) involving 9,484 adult participants with a mean follow-up of 3.7 years. The group with interventions involved lower targets for SBP/DBP (135/85 mmHg or less) compared with the standard target group for SBP (140–160 mmHg)/DBP (90–100 mmHg or less). Participants were adults with documented hypertension and adults receiving treatment for hypertension with a history of myocardial infarction, stroke, chronic peripheral vascular occlusive disease, or angina pectoris. They found that there was little to no difference in total mortality between the two BP-target groups. They also found that there might be little to no difference in total CVD between them.

Nawata and Kimura (36) evaluated the effects of BP on increases in medical costs using the power transformation tobit models and a dataset consisting of 175,123 medical checkups and 6,312,125 receipts from 88,211 individuals obtained from April, 2013 to March 2016. They found a negative relationship between SBP and medical costs. Using the same dataset, Nawata and Kimura (37) analyzed the effects of BP on medical costs and on the probability of having a history of HD. The dataset was divided into subgroups based on whether the patients had diabetes and took antihypertension medications. They could not find any evidence that higher SBP increased medical costs or the probability of having HD. Nawata et al. (38) analyzed the effects of BP and antihypertensive medications on the probability of having HD in the subsequent year. Their dataset, containing 83,287 medical checkup and treatment records obtained from 35,504 individuals in 5 fiscal years, was analyzed by probit models. They could not find any evidence that higher SBP increased the probability of undergoing treatment for HD. However, DBP increased the probability in most of the models. They also found that taking antihypertensive medications actually increased the probability of undergoing treatment for HD. The results of these studies did not support the 2017 ACA/AHA guideline. Nawata (39) also suggested ischemic stroke as a potential side effect of antihypertensive medications. Vinyoles et al. (40) conducted a study focused on CVD mortality and morbidity using the Cox regression analysis. Their study included 3,907 patients without CVD, recruited beginning in July 2004, with an average follow-up period of 6.6 years. They reported that office BP lost its significant association, but the association of ambulatory BP with CVD mortality and morbidity remained significant in the fully adjusted model.

The results of the former and latter groups of studies were quite different. Nawata et al. (41) pointed out the potential sources of the incongruities. First, publication biases, conflicts of interest, and differences in termination (or endpoint) may have played a role. Second, for meta-analyses and systematic reviews, the selection criteria of the studies may not be rigorous. Moreover, the selected studies usually use different samples, methodologies and factors affecting CVD, and it is difficult to adjust for these differences. Third, the effects of age might not be removed when the age intervals of cohorts in cohort studies are too large. In this case, BP works as a proxy variable for age. Finally, although the Cox proportional hazard model (and modified models such as the Cox frailty model) is used in many analyses, it may not give correct results when time-dependent variables exist among the covariates (38).

## Data and models

### Data

In Japan, most employees age 40 or older undergo mandatory medical checkups at least once a year due to the Industrial Safety and Health Act. Employees under age 40 and family members of employees may undergo medical checkups on a voluntary basis. The JMDC Claims Database is the nationwide health information database, which collects data from various health insurance societies in Japan. It contains the results of 13,157,681 medical checkups obtained from 3,233,271 individuals between January 2005 and September 2019. Note that we focused on HD; other types of CDV are not analyzed in this study. In the database, various types of heath information of individuals containing histories of HD are included. To delineate the relationships among BP, antihypertensive medications, and HD more clearly, we choose individuals who had no HD histories at year t and had data (either positive or negative) reflecting HD at year *t* + 1 (i.e., the following year). 6,773,464 observations satisfied these criteria, and 35,551 observations or 0.52% had HD at year *t* + 1 among the total observations.

**Figure 1:**
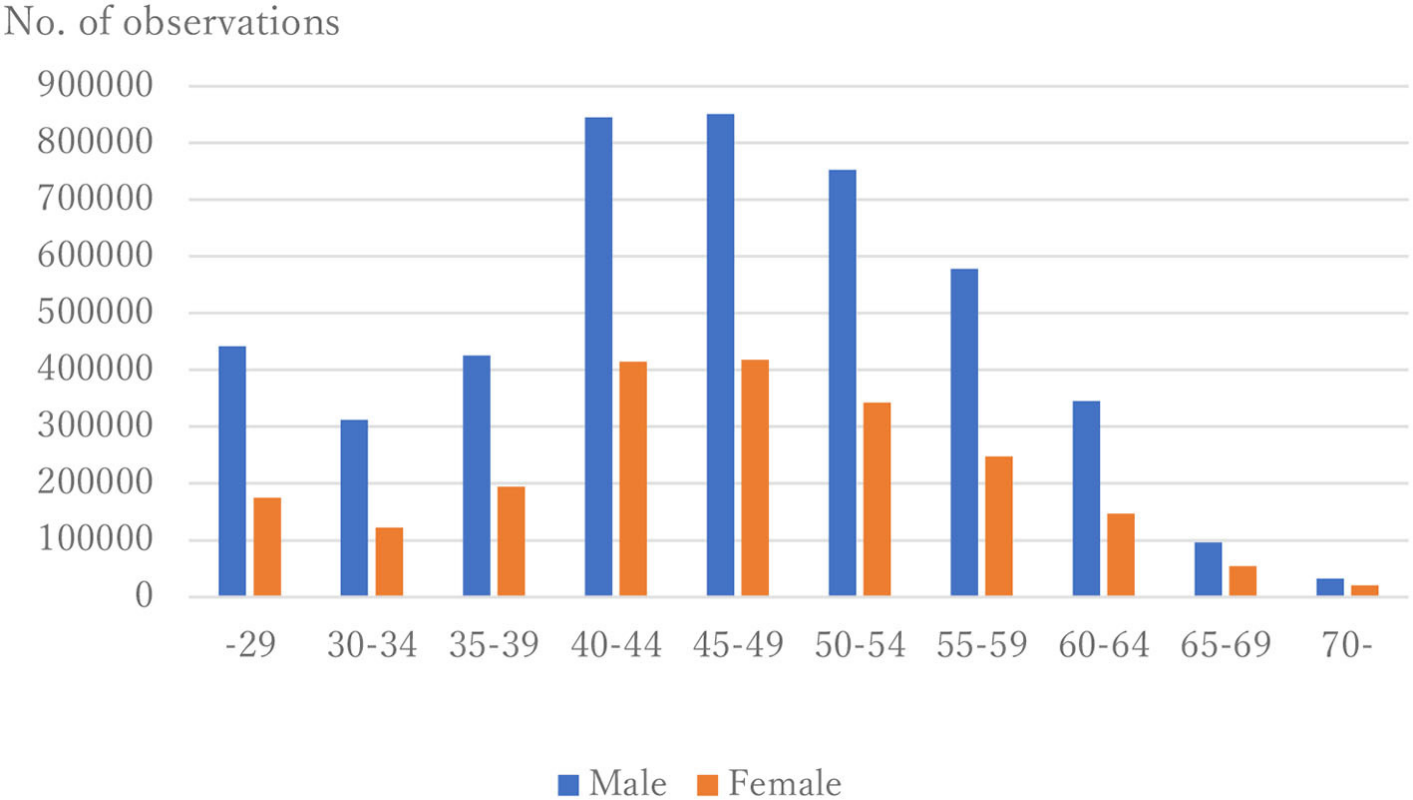
Distribution of observations by age and gender.

**Figure 2:**
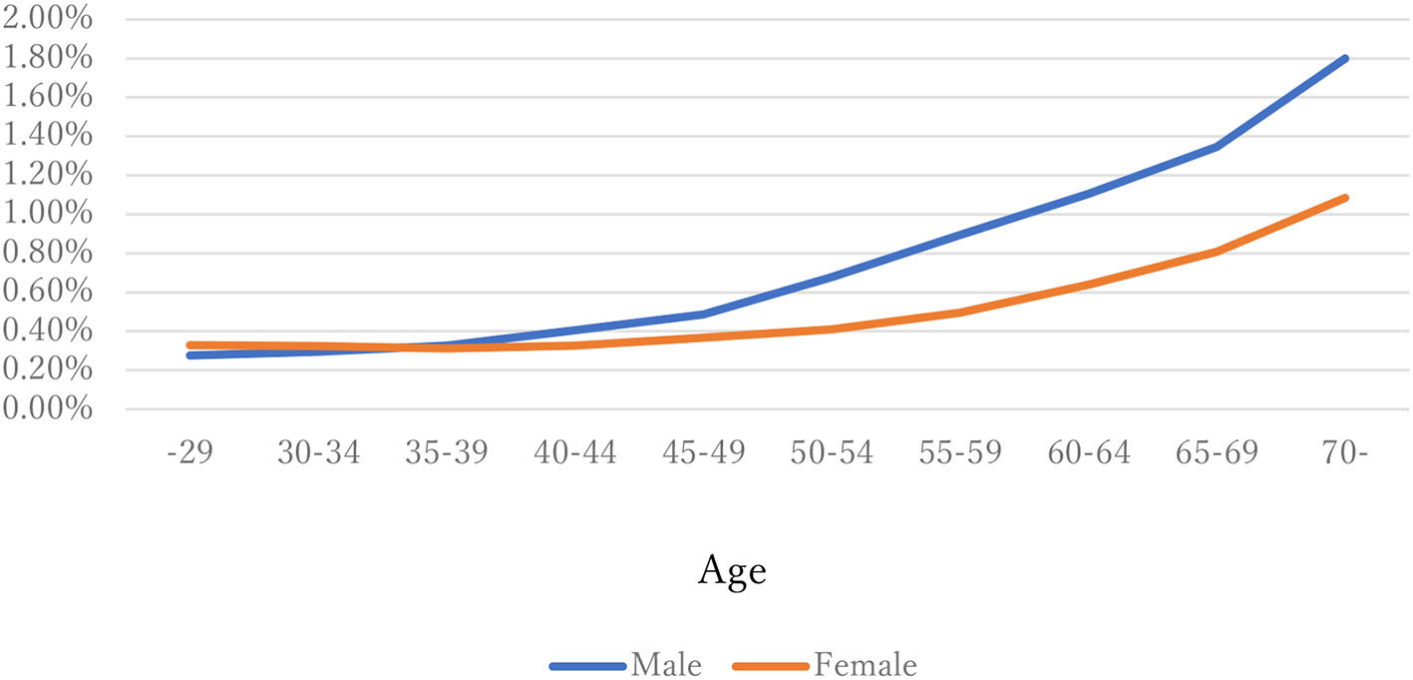
The percentages of having HD by next year by age and gender.

**Figure 3:**
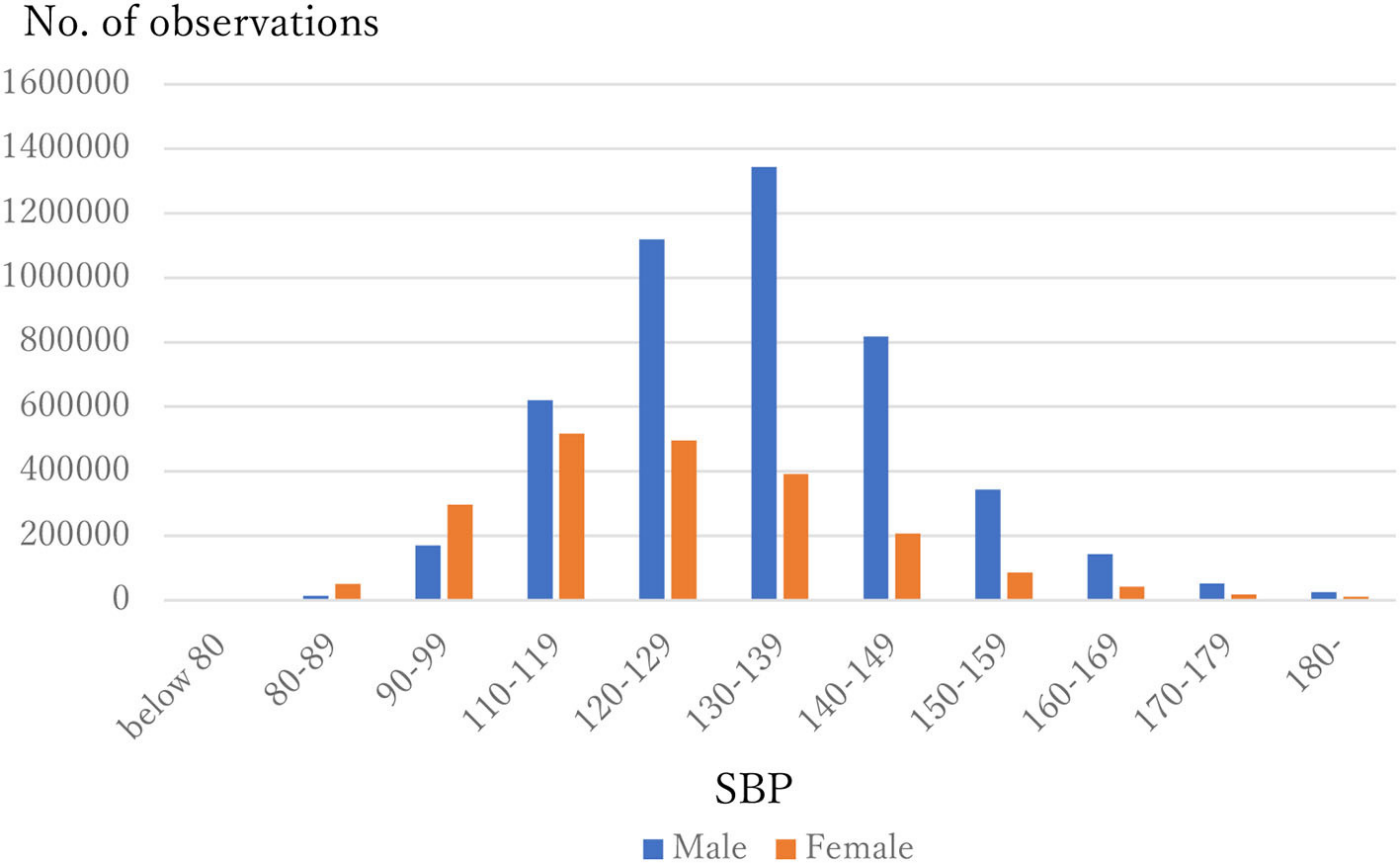
The distribution of SBP by gender.

**Figure 4:**
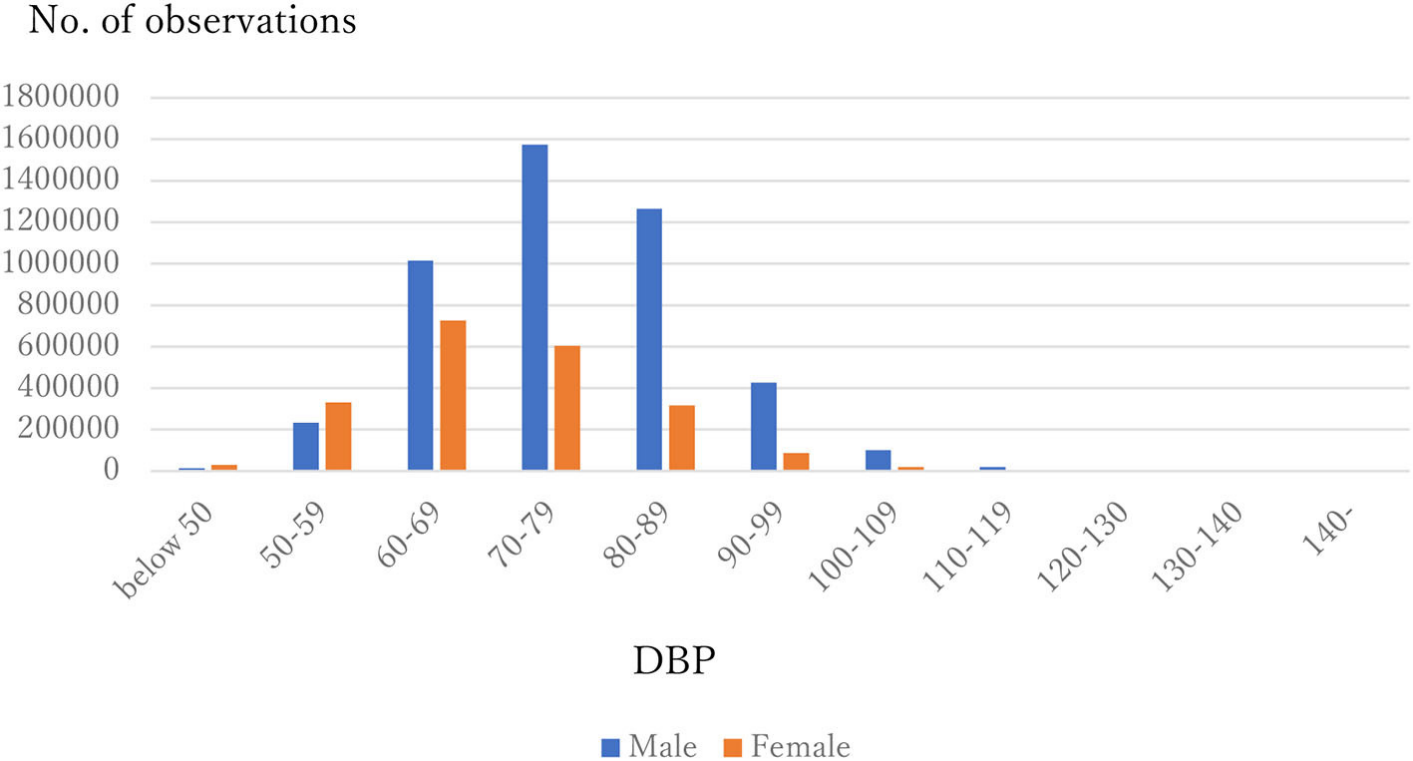
Distribution of DBP by gender.

**Figure 5:**
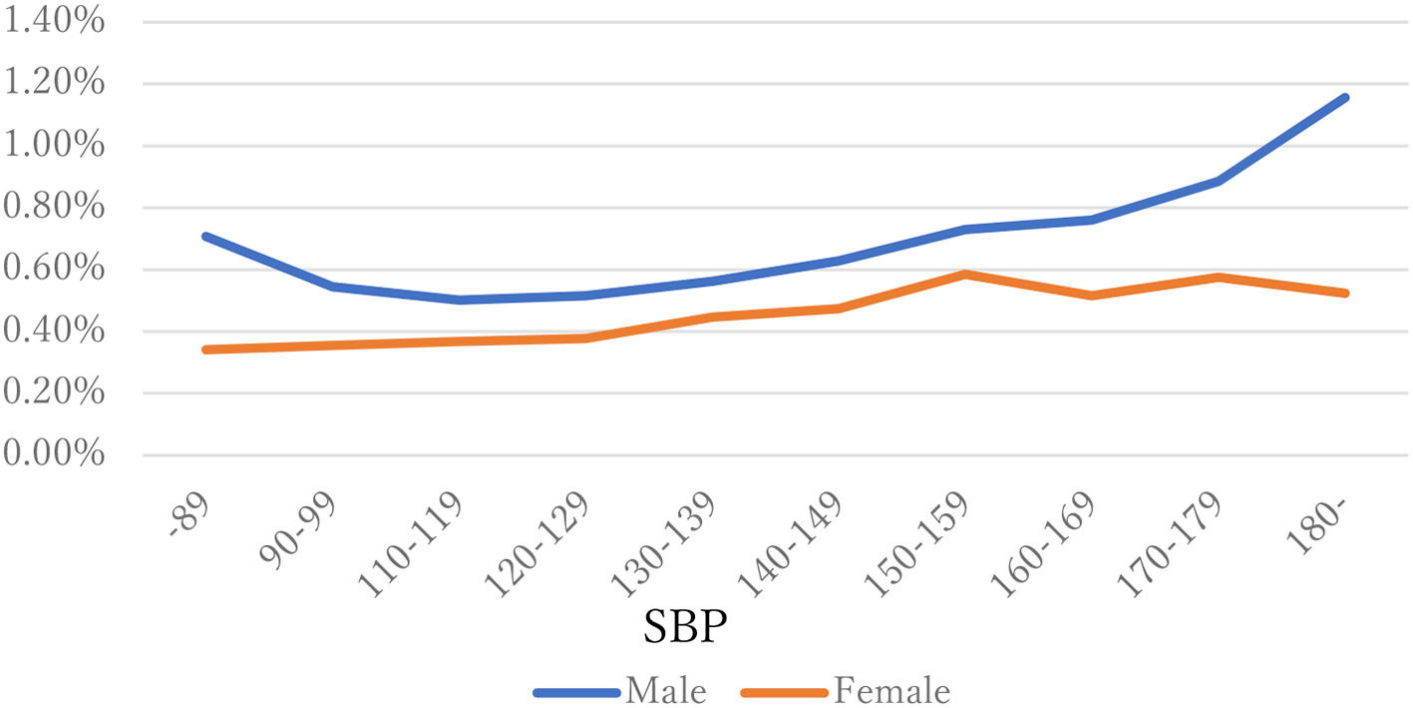
SBP and percentages having HD by next year by gender.

The distribution of observations by age and gender is given in Figure 1. As these were employment-based health checkups, few data for those age >70 years are included. The percentages of observations that had HD at their next annual checkup are given in Figure 2 and are categorized by age and gender. For males, the percentage is almost constant until age 40. After that it increases until it reaches 1.80% at age 70 or over. For females, the percentage is almost constant until age 50 then increases until it reaches 1.08% at age 70 or over. The percentages are similar for males and females until age 40, but the percentage of males increases much faster than that of females after that. Figures 3, 4 show the distributions of SBP and DBP. There are large differences between males and females. Under the 140/90 mmHg criterion, 12.2% of males and 7.4% of females would be diagnosed with SBP hypertension, and 11.8% of males and 5.2% of females would be diagnosed with DBP hypertension. However, under the 130/80 criterion, 29.8% of males and 17.2% of females would be diagnosed with SBP hypertension, and 39.1% of males and 20.2% of females would be diagnosed with DBP hypertension. Figure 5 shows the percentages of observations that had HD by the next year by both age and SBP. The percentage clearly increases if SBP is higher than 130 mmHg, especially for males; however, the increment is not very clear for females.

### Causality problem of hypertension and HD

We analyze the probability of an individual who had no history of HD at year t and did develop HD by year *t* + 1 using probit models. Similar models were used by Nawata et al. (38), Nawata (39), and Cohen et al. (42). The previous studies (43–46) suggest the possibility that HD might cause hypertension (rather than hypertension causing HD). One key advantage of the models in this analysis is that the direction of causality is certain, since we used data including SBP and DBP from the year prior to the occurrence of HD. First, we considered models with basic factors of individuals as covariates, and then examined models with various health-related factors determined at medical checkups.

### Models with basic factors

Among 6,773,464 observations, 35,551 observations or 0.52% had HD the following year. Let *HD*_*t*+1_ be a dummy variable if an individual had HD by year *t* + 1. (Note that from the definition of the variable, *HD*_*t*_ = 0.) First, we consider the following simple model.

Model A:


(1) $$P\left[HD_{t+1}=1|HD_{t}=0\right]=\Phi (\beta_{1}+\beta_{2}SBP_{t}),$$

where Φ is the distribution function of the standard normal distribution, and *SBP*_*t*_ is SBP at year t. The results of the estimation are given under “Model A” in Table 1. The estimate of *SBP*_*t*_ is positive. The *t-*value is 30.67 and is significant at any reasonable level. From this result, SBP seems a very important variable for HD. However, if we add age, gender and body mass index (BMI), the importance of SBP is diminished. We consider the following model.

**Table 1:** Results of estimation: models A and B.

**Variable**	**Model A**			**Model B**		
	**Estimate**	**SE**	**t-value**	**Variable**	**Estimate**	**t-value**
Constant	−2.9781	0.0139	−214.522	−3.4366	0.0178	−193.0796
*SBP*	0.003443	0.000112	30.674	−0.000205	0.00013	−1.6164
*Age*				0.0137	0.0002	73.2498
*Female*				−0.1037	0.0044	−23.5778
*BMI*				0.0117	0.000526	22.2435
log Likelihood		−221630.8			−218185.0	
No. of Cases	0:673713; 1:35551; total:6773464			0:6733632; 1:35532; total:676914		

SE, Standard error.

Model B:


(2) $$\begin{array}{l} P\left[HD_{t+1}=1\right]=\Phi (\beta_{1}+\beta_{2}SBP_{t}+\beta_{3}Age_{t}+\beta_{4}Female_{t}\\ +\beta_{4}BMI_{t}), \end{array}$$

where *Age*_*t*_ is the age of the individual at year t, *Female*_*t*_ is the dummy variable that takes 1 if the individual is female and 0 if the individual is male, and *BMI*_*t*_ is the value of BMI. The results of the estimation are given under “Model B” in Table 1. The estimate of *SBP*_*t*_ becomes negative (though insignificant.).

Next, we consider a model where the effect of age is expressed by dummy variables of 10-year age intervals.

Model C:


(3) $$\begin{array}{l} P\left[HD_{t+1}=1\right]=\Phi (\beta_{1}+\beta_{2}SBP_{t}+\beta_{3}Age60_{t}+\beta_{4}Age70_{t}\\ +\beta_{6}Female_{t}+\beta_{6}BMI_{t}), \end{array}$$

where *Age*60_*t*_*and Age*70_*t*_ are dummy variables such that *Age*60_*t*_=1 if an individual is age 60-69 and 0 otherwise, and *Age*70_*t*_=1 if an individual is age 70 or over and 0 otherwise. The results of the estimation are given in Table 2. In this case, the association of *SBP*_*t*_ with HD at year *t* + 1 becomes positive and significant at the 1% level (the *t-*value is 5.88).

**Table 2:** Results of estimation: model C.

**Variable**	**Model C**		
	**Estimate**	**SE**	***t*-value**
Constant	−2.9726	0.0166	−178.6692
*SBP*	0.000740	0.000126	5.8848
*AGE60*	0.2732	0.0052	52.7683
*AGE70*	0.4421	0.0142	31.1980
*Female*	−0.0937	0.0044	−21.3846
*BMI*	0.0132	0.0005	25.5356
log Likelihood		−221970.9	
No. of cases	0:6733632; 1:35532; total:676914		

SE, standard error.

### Models containing various health factors

Next, models containing various health factors as covariates are considered.

Model D:


(4) $$\begin{array}{l} P\left[HD_{t+1}=1\right]=\Phi (\beta_{1}+\beta_{2}Age+\beta_{3}Female+\beta_{4}t1\\ +\beta_{5}BMI+\beta_{6}\;SBP\\ +\beta_{7}DBP+\beta_{8}HDL+\beta_{9}LDL+\beta_{10}Triglyceride+\beta_{11}ALT\\ +\beta_{12}AST+\;\beta_{13}GGP\\ +\beta_{14}U\_Sugar+\beta_{15}U\_Protein+\beta_{16}Weight\_1\\ +\beta_{17}Weight\_20\;+\beta_{18}Eat\_Fast\\ +\beta_{19}Late\_Supper+\beta_{20}No\_Breakfast+\beta_{21}Exercise\\ +\beta_{22}Activity+\beta_{23}Walk\_Fast\\ +\beta_{24}Sleep+\beta_{25}Alcohol\_Freq+\beta_{26}Alcohol\_Amount\\ +\beta_{27}Smoke+\beta_{28}M\_\;Antihypertensive\\ +\beta_{29}M\_Glucose+\beta_{30}M\_Cholestrol). \end{array}$$

To avoid unnecessary notation, the subscript *t* is omitted in covariates; all covariates can be assumed to be measured at year *t*. The definitions and summaries of the covariates are given in Tables 3, 4. The results of estimation are given under “Model D” in Table 5. The estimates of *Age* and *Female* are highly significant and very important variables. The estimates of *Family, HDL, LDL, ALT* and *Sleep* are negatively associated with HD at year *t* + 1 and significant at the 1% level. The estimates of *AST, HbA1c, U_Protein, Weight_1, M_ Anihypertensive* and *M_Cholesterol* were positive and significant at the 1% level. Despite the very large size of the sample, the estimates of other variables, including *SBP* and *DBP*, were not significant even at the 5% level. It is a somewhat surprising that the estimate of *M_Antihypertensive* was positive and highly significant (the *t-*value is 29.14).

**Table 3:** Definition of covariates.

**Variable**	**Definition**	**Variable**	**Definition**
*Female*	1:female; 0:male	*Weight_1*	1: weight changed by 3 kg or more in a year; 0:otherwise
*Family*	1:family member; 0: otherwise	*Weight_20*	weight increased by 10 kg or more from age 20
*t1*	time trend =year-2004	*Eat_fast*	1: eating faster than other people; 0: otherwise
*BMI*	body mass index =weight (kg)/ height (m)^2^	*Late_supper*	1: eating supper within 2 hours before bedtime three times or more in a week; 0: otherwise,
*SBP*	systolic blood pressure, mmHg	*No_breakfast*	1: not eating breakfast three times or more in a week; 0: otherwise
*DBP*	diastolic blood pressure, mmHg	*Exercise*	1: doing exercise for 30 minutes or more twice or more in a week for more than a year; 0 otherwise
*HDL*	high-density lipoprotein cholesterol blood, mg/dL	*Activity*	1: doing physical activities (walking or equivalent) for 1 hour or more daily, 0: otherwise
*LDL*	low-density lipoprotein cholesterol, mg/dL	*Speed*	1: walking faster than other people of a similar age and the same gender; 0: otherwise
*Triglyceride*	mg/dL	*Sleep*	1: sleeping well; 0: otherwise
*ALT*	alanine aminotransferase, U/L	*Alcohol_freq*	0: not drinking alcoholic drinks, 1: sometimes, 2: every day
*AST*	aspartate aminotransferase, U/L	*Alcohol_amount*	0: not drinking; 1: drinking less than 180 ml of Japanese sake wine (with an alcohol percentage of about 15%) or equivalent alcohol in a day when drinking; 2: drinking 180–360 ml; 3: drinking 360–540 ml; 4: drinking 540 ml or more,
*GGP*	γ-glutamyl transferase, units per liter	*Smoke*	1: smoking; 0: otherwise
*B_Sugar*	blood sugar, mg/dL	*M_Antihypertensive*	1:taking antihypertensive medications , 0: otherwise
*HbA1c*	hemoglobin A1c, %	*M_Glucose*	taking medications to control glucose levels (including insulin injections)
*U_Protein*	1:undetected, 2: around 15 mg/dL, 3: around 30 mg/dL, 4: around100 mg/dL and 5: around 250 mg/dL or over	*M_Cholestrol*	taking medications to control cholesterol or *triglycerides*
*U_Sugar*	1 :undetected, 2: around 50 mg/dL, 3: around 100 mg/dL, 4: around 250 mg/dL and 5: around 500 mg/dL or over		

**Table 4:** Summary of covariates.

**Variable**	**Summary**		**Variable**	**Summary**
	**Average**	**SD**		
*Age*	47.93	9.49	*Weight*_1	1:26.1%, 0:74.2%
*Female*	1: 38.2%; 0: 61.8%		*Weigh*t_20	1:35.2%; 0:64.8%
*Family*	1: 22.1%; 77.9%		*Eat_fast*	1:32.4%; 67.6%
t1	11.1	2.05	*Late_supper*	1:32.3%; 0:67.7%
*BMI*	23.0	3.65	*No_breakfast*	1:17.8%; 0:82.2%
*SBP*	119.9	16.22	*Exercise*	1:21.6%; 0:78.4%
*DBP*	74.5	11.81	*Activity*	1:34.9%; 0:75.1%
*HDL*	63.50	16.80	*Speed*	1:45.0%; 0:55.0%
*LDL*	121.9	30.84	*Sleep*	1:59.0%; 0:40.1%
*Triglyceride*	108.3	85.96	*Alcohol_freq*	0:40.6%, 1:34.0%; 2:25.3%
*ALT*	23.2	17.70	*Alcohol_amount*	0:40.6%; 1:22.1%; 2:22.8%; 3:10.7%; 4:3.8%
*AST*	22.3	10.64	*Smoke*	1:25.6%; 0:74.4%
*GGP*	38.2	45.49	*M_Antihypertensive*	1:11.18%; 0:88.82%
*B_Sugar*	95.5	18.18	*M_Glucose*	1:3.21%; 0:96.79%
*HbA1c*	5.54	0.60	*M_Cholestrol*	1:7.46%; 0:92.54%
*U_Protein*	1:89.44%; 2:7.68%; 3:2.25%; 4:0.53%; 5:0.14%		
*U_Sugar*	1:97.86%; 2:0.46%; 3:0.53%; 4:0.39%; 5:0.77%		

SD, Standard deviation.

**Table 5:** Results of estimation: models D and E.

**Variable**	**Model D**			**Model E**		
	**Estimate**	**SE**	***t*-value**	**Estimate**	**SE**	***t*-value**
Constant	−3.2417	0.0459	−70.583	−3.5631	0.0432	−82.567
*Age*	0.0130	0.0004	35.344	0.0163	0.0004	46.581
*Female*	−0.0967	0.0096	−10.078	−0.0975	0.0096	−10.211
*Family*	−0.0294	0.0107	−2.739	−0.0286	0.0107	−2.677
*t1*	0.0001	0.0014	0.063	0.00002	0.0014	−0.013
*BMI*	0.0007	0.0011	0.691	0.0059	0.0011	5.533
*SBP*	−0.000173	0.000290	−0.596	0.000385	0.000289	1.334
*DBP*	−0.000102	0.000407	−0.251	−0.000037	0.000406	−0.090
*HDL*	−0.0012	0.0002	−5.627	−0.0015	0.0002	−6.690
*LDL*	−0.0005	0.0001	−4.684	−0.0011	0.0001	−10.962
*Triglyceride*	−0.000043	0.00004	−1.192	−0.00004	0.000036	−1.172
*ALT*	−0.0011	0.0003	−3.727	−0.00091	0.00028	−3.252
*AST*	0.0011	0.0004	2.865	0.00113	0.00039	2.880
*GGP*	0.00008	0.00007	1.220	0.00015	0.00007	2.279
*B_Sugar*	0.0001	0.0002	0.451	−0.0071	0.0066	−1.080
*HbA1c*	0.0257	0.0069	3.710	0.0370	0.0066	5.607
*U_Protein*	0.0389	0.0053	7.374	0.0511	0.0053	9.736
*U_Sugar*	−0.0086	0.0066	−1.312	−0.0052	0.0078	−0.666
Weight_1	0.0648	0.0066	9.805	0.0650	0.0066	9.865
*Weight_20*	0.0137	0.0072	1.923	0.0187	0.0071	2.639
*Eat_fast*	0.0049	0.0062	0.786	0.0084	0.0062	1.362
*Late_supper*	0.0100	0.0065	1.539	0.0067	0.0064	1.037
*No_breakfast*	0.0102	0.0080	1.274	0.0055	0.0080	0.694
*Exercise*	0.0012	0.0072	0.163	−0.0002	0.0072	−0.031
*Activity*	0.0048	0.0064	0.757	0.0037	0.0064	0.580
*Speed*	−0.0063	0.0059	−1.066	−0.0115	0.0059	−1.940
*Sleep*	−0.0452	0.0059	−7.700	−0.0444	0.0059	−7.593
*Alcohol_freq*	−0.0096	0.0055	−1.740	−0.0096	0.0055	−1.761
*Alcohol_amount*	0.0064	0.0037	1.722	0.0084	0.0037	2.286
*Smoke*	−0.0116	0.0069	−1.679	−0.0226	0.0069	−3.283
*M_ Antihypertensive*	0.2318	0.0080	29.135			
*M_Glucose*	0.0181	0.0151	1.201			
*M_Cholestrol*	0.0868	0.0093	9.368			
Log Likelihood		−69378.8			−69730.6	
No. of cases	0:2844824; 1:15705; total 2860529			0:2846056;1:15713 total:2861769		

SE, standard error.

Taking medications affects health factors and health factors affect whether an individual takes medications or not. For example, taking antihypertensive medications affects BP, and an individual with high BP levels is more likely to take antihypertensive medications. Therefore, the following model (statistically, the reduced form) was also considered.

Model E:


(5) $$\begin{array}{l} P\left[HD_{t+1}=1\right]=\Phi (\beta_{1}+\beta_{2}Age+\beta_{3}Female+\beta_{4}t1\\ +\beta_{5}BMI+\beta_{6}\;SBP\\ +\beta_{7}DBP+\beta_{8}HDL+\beta_{9}LDL+\beta_{10}Triglyceride+\beta_{11}ALT\\ +\beta_{12}AST+\;\beta_{13}GGP\\ +\beta_{14}U\_Sugar+\beta_{15}U\_Protein+\beta_{16}Weight\_1\\ +\beta_{17}Weight\_20\;+\beta_{18}Eat\_Fast\\ +\beta_{19}Late\_Supper+\beta_{20}No\_Breakfast+\beta_{21}Exercise\\ +\beta_{22}Activity+\;\beta_{23}Walk\_Fast\\ +\beta_{24}Sleep+\beta_{25}Alcohol\_Freq+\beta_{26}Alcohol\_Amount\\ +\beta_{27}Smoke). \end{array}$$

The results of estimation are given under “Model E” in Table 5. The estimation results are very similar to those of Model D except for *BMI*. As with the previous model, the estimates of *Age* and *Female* were highly significant. The estimates of *Family, HDL, LDL, ALT, Sleep, Speed* and *Smoke* were negative and significant at the 1% level. The estimates of *AST, HbA1c, U_Protein, Weight_1* and *Weight_20* were positive and significant at the 1% level, and those of *GGP* and *Alcohol_Amount* were positive and significant at the 5% level. For *BMI* the estimate was not significant at the 5% level in Model D, but it became positive and significant at the 1% level in Model E. The estimates of other variables including *SBP* and *DBP* were not significant at the 5% level.

## Discussion

**Figure 6:**
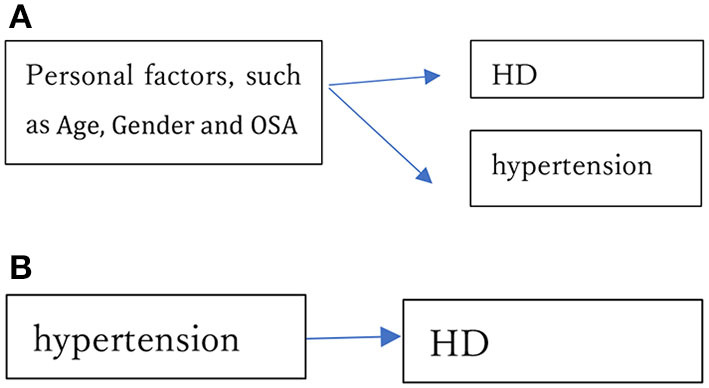
**(A)** Personal factors, such as age, gender and obstructive sleep apnea (OSA), simultaneously cause hypertension and HD. **(B)** Hypertension directly causes HD.

The estimate of SBP was positive and highly significant in Model A. Interestingly, it became negative (though not significantly; *p-*value, 0.11) in Model B, which includes age, gender and BMI as covariates. For analyses of the relation between BP and HD, it is very important to consider all necessary factors. We can consider two possibilities. One is that personal factors (such as age and gender) simultaneously and similarly impact hypertension and HD but that hypertension is not a direct risk factor of HD as shown in Figure 6A. For instance, Collen et al. (47) demonstrated that obstructive sleep apnea (OSA) is frequently linked to most HD, and that OSA patients were at substantial risk of developing hypertension, but that treatment of hypertension did not prevent HD in those individuals. If OSA is the cause of HD, it is necessary to treat OSA, not hypertension. Similarly, Teixeira et al. (48) evaluated the effect of occupational exposure to noise on IHD and hypertension. Although they judged the existing body of evidence from human data to provide “limited evidence of harmfulness”, treatment of hypertension would not reduce the risk of IHD if the noise causes IHD and hypertension simultaneously. In our analysis, the estimates of BP levels became insignificant when key variables were included. In many cases, they were significant if the key variables were not included. In other words, the relations that have been observed may be spurious.

The other possibility is that hypertension directly causes HD as shown in Figure 6B. Treatment of hypertension helps to prevent HD in this case, and hypertension would remain significantly associated even if various other factors representing health conditions were included in the analysis. Nawata (49) showed that there are strong relations among age, gender and BP. The estimation results of Models A and B strongly suggest the former possibility; that is, that the relationship commonly observed between hypertension and HD might be spurious. To evaluate whether a study has shown a true relationship between BP levels and HD risk, one must consider whether all key factors have been included, especially those key factors that might cause hypertension and HD simultaneously.

Another problem is that the length of the age interval may or may not be properly chosen, especially in cohort studies. Nawata (49) reported that SBP increased by about 4.6 mmHg from age 50 to 60. In Model C, the estimate of SBP becomes positive and significant at the 1% level. Thus, SBP might work as a proxy variable for age if the age interval is 10 years or longer. In other words, the 10-year age interval may be too coarse as a variable, such that it might be necessary to reevaluate studies with long age intervals.

In Models D and E, the estimates of SBP and DBP become insignificant even at the 5% level, despite the very large sample sizes (2,860,529 and 2,861,769 for Models D and E, respectively). The estimates of both SBP and DBP are negative in Model D and the estimate of DBP is negative in Model E. This means that we could not find any evidence that hypertension is a risk factor for HD in this study.

Perhaps the most surprising of our findings, the estimate of *M_ Antihypertensive* is positive and highly significant (the *t-*value is 29.14). This means that all else being equal (including SBP and DBP), those taking antihypertensive medications are more likely, not less likely, to develop HD. Colantonio et al. (50) analyzed the data for Black and White participants in the REGARDS (REasons for Geographic and Racial Differences in Stroke) study to evaluate the 2017 ACC/AHA guideline. They reported that the CVD rates per 1,000 person-years without the recommended antihypertensive medications were much lower than those who took those medications. Their findings coincide with the estimation result of Model D. Musini et al. (51) reviewed trials of pharmacotherapy for hypertension. They admitted that “trials in the very elderly used higher doses of more antihypertensive drugs and showed a trend toward increased total mortality.” They also reported a significant increase in withdrawals from the trials due to the adverse effects of drugs.

There are several possible interpretations of this result. One is that individuals taking antihypertensive medications go to hospitals or clinics more often than those without medications, and their HD is thus more likely to be found. The second is that there exist unobserved risk factors, and taking antihypertensive medications works as a proxy variable for these risk factors. The third is that taking antihypertensive medications is itself a risk factor for HD. For the first interpretation, evaluating the health conditions of those who present at hospitals or clinics frequently is important. For the second, it may be necessary to find out the unobserved risk factors. For the third one, further studies are needed on the risks of antihypertensive medications, and they should be prescribed with caution.

Apart from BP levels, cholesterol levels are important factors. The WHO (52) and British Hart Foundation (53) have established that elevated cholesterol levels increase the risks of heart disease. The CDC (54) has labeled HDL “good” cholesterol and LDL “bad” cholesterol. However, the estimation results of Models D and E suggest something different. Higher HDL and LDL levels appear to reduce the risk of HD in our sample of data from Japanese workers. The CDC advises (55) Americans to “limit foods high in saturated fat. Saturated fats come from animal products...” However, Sauvaget et al. (56) reported that a higher consumption of animal fat and cholesterol was associated with a reduced risk of death from cerebral infarction in Japan. A similar phenomenon might be occurring for HD.

## Conclusion

In this paper, the risk factors of HD were analyzed using the JMDC Claims Database which included 13,157,681 medical checkups obtained from 3,233,271 individuals in Japan. Data from checkups of individuals who had no history of HD at year t and had data (either positive or negative) regarding HD at year *t* + 1 (i.e., the following year) were selected, and the probabilities that they developed HD by year *t* + 1 were analyzed using the probit models. There was a strong relation between SBP and HD when the relation of just those two variables was evaluated (Model A). However, when age, gender and BMI were included in the model (Model B), no significant relation was observed; suggesting that the relation between hypertension and HD might be spurious. Moreover, when long age intervals were used in a model (e.g., 10-year intervals), BP appeared to work as a proxy variable for age (Model C). In the models containing numerous covariates representing various health-related conditions and habits (Models D and E), the estimates of SBP and DBP were insignificant even at the 5% level. The estimate of the effect of taking antihypertensive medications was highly significant and positively related to HD risk, suggesting the need for further reevaluation of current preventative health practices regarding BP and HD.

Other than BP, cholesterol levels are important factors. It is generally held that elevated cholesterol levels increase the risk of heart disease. HDL is called a “good” cholesterol and LDL a “bad” cholesterol. However, the results of this study suggest that higher HDL and LDL levels are all associated with reduced risk of HD. Thus, the relation between cholesterol levels and HD may also need to be reevaluated.

### Limitations

The dataset is observatory and includes mainly working-generation individuals. It does not include those aged 76 or over. Data from elderly people should be collected and included in further analyses. The types of antihypertensive medications used were not analyzed. The dataset was obtained only in Japan and the results might differ in other countries.

## Data availability statement

Publicly available datasets were analyzed in this study. This data can be found here: https://phm.jmdc.co.jp/en/database.

## Ethics statement

The studies involving human participants were reviewed and approved by Institutional Review Boards of Hitotsubashi University. Written informed consent for participation was not required for this study in accordance with the national legislation and the institutional requirements.

## Author contributions

The author confirms being the sole contributor of this work and has approved it for publication.

## Conflict of interest

The author declares that the research was conducted in the absence of any commercial or financial relationships that could be construed as a potential conflict of interest.

## Publisher's note

All claims expressed in this article are solely those of the authors and do not necessarily represent those of their affiliated organizations, or those of the publisher, the editors and the reviewers. Any product that may be evaluated in this article, or claim that may be made by its manufacturer, is not guaranteed or endorsed by the publisher.
